# Understanding how young African adults interact with peer-generated sexual health information on Facebook and uncovering strategies for successful organic engagement

**DOI:** 10.1186/s12889-021-12165-x

**Published:** 2021-11-24

**Authors:** Emmanuel Olamijuwon, Odimegwu Clifford, Visseho Adjiwanou

**Affiliations:** 1 Demography and Population Studies Programme, Schools of Public Health and Social Sciences, University of the Witwatersrand, Johannesburg, South Africa; 2 Department of Statistics and Demography, School of Social Science, University of Eswatini, Kwaluseni Campus, Kwaluseni, Eswatini; 3 Population and Health Research Group, School of Geography and Sustainable Development, University of St Andrews, Fife, UK; 4 Department of Sociology, Université du Québec à Montréal, Montreal, Canada

**Keywords:** Sexuality education, Sexual health, Adolescents, Young adults, Social media, Facebook, Content analysis

## Abstract

**Background:**

The use of social media for sexual health communication is gaining intense discussion both globally and in Africa. Despite this reality, it remains unclear whether and how young African adults use digital innovations like social media to access sexual health information. More importantly, the unique properties of messages that increase message reach and propagation are not well understood. This study aims to fill the gaps in scholarship by identifying post features and content associated with greater user engagement.

**Methods:**

We analyzed a corpus of 3533 sexual and reproductive health messages shared on a public Facebook group by and for young African adults between June 1, 2018, and May 31, 2019, to understand better the unique features associated with higher engagement with peer-generated sexual health education. Facebook posts were independently classified into thematic categories such as topic, strategy, and tone of communication.

**Results:**

The participants generally engaged with posts superficially by liking (x̃ = 54; x̄ = 109.28; σ = 159.24) rather than leaving comments (x̃ = 10; x̄ = 32.03; σ = 62.65) or sharing (x̃ = 3; x̄ = 11.34; σ = 55.12) the wallposts. Messages with fear [IRR:0.75, 95% CI: 0.66–0.86] or guilt [IRR:0.82, 95% CI: 0.72–0.92] appeals received a significantly lower number of reactions compared to neutral messages. Messages requesting an opinion [IRR:4.25, 95% CI: 3.57–5.10] had a significantly higher number of comments compared to status updates. The use of multimedia and storytelling formats were also significantly associated with a higher level of engagement and propagation of sexual health messages on the group.

**Conclusion:**

Young adults in our sample tend to superficially interact with peer-communicated sexual health information through likes than engage (comments) or propagate such messages. Message features that increase engagements and propagation of messages include multimedia and engaging styles like storytelling. Our findings provide valuable insight and pave the way for the design of effective and context-specific sexual health information use of features that attract young African adults.

## Background

Sexual behaviour among young African adults presents significant public health concerns, in part because young people continue to have high rates of sexually transmittable infections, including Human Immunodeficiency Virus (HIV) [1], an unmet need for family planning, and low efficacy for condom use [2–6]. On the other hand, sexuality also forms an integral aspect of health and wellbeing, especially in the context of comprehensive sexuality education and unrestricted access to health services [7, 8]. As a result, there is an increasing need to provide young people with comprehensive sexual health education to enhance intimacy and the realization of relationship goals and empower them to make informed decisions about their sexual and reproductive health [1, 9–12]. Today, several African countries have scaled up the dissemination of sexual health information using mass media, bulk messaging platforms, and in-school training, among many others [1, 13–18].

There is increasing advocacy for experimenting with alternative and new methods to sexual health promotion, particularly those that provide safe spaces to engage and provide young people with comprehensive sexuality education [19]. Accordingly, social media internet sites like Facebook, Twitter and YouTube are quickly replacing traditional forms of communication because they offer individual users rapid transference of ideas and opinions through a relatively low-cost and user-friendly network [20, 21]. More importantly, social media platforms offer new channels for health communication that, when matched to the needs and preferences of the target audience, can increase the chances of programming success [22]. This could be because, unlike other methods of disseminating sexual health information, social media sites offer a multidirectional communication model in which audience members participate actively in discussions and share their knowledge and experiences, rather than being passive recipients of sexual health information [23, 24]. These multidirectional engagements ultimately offer a promising opportunity to understand young people’s perspectives and identify dominant stereotypes and misinformation while correcting misinformation and addressing concerns that emerge from such interactions [25, 26].

Furthermore, the extensive reach of social networking sites like Facebook and their interaction functions offer huge potentials in delivering health promotion messages [27, 28]. In fact, a recent scoping review demonstrated that sexual health education delivered via social media effectively increases health knowledge, awareness and ultimately motivates behavioural change [29]. Young adults alike are receptive to this communication channel as multiple studies have shown that adolescents and young adults prefer to access sexual and reproductive health information from social media, particularly Facebook [20, 30, 31]. As young people use these forms of new media, including social media, there may be new opportunities to listen to and engage with young African adults about sexual health issues [32].

Nevertheless, health information is only effective if it reaches and engages with its target audience. Interactive health education strategies that leverage the full potential of social media by encouraging participation and engagement rather than providing a one-way flow of information are reported to have greater potential to enhance behaviour change [33, 34]. This is important because engagement metrics such as reactions (favourites), comments (replies) and shares (retweets) provide rudimentary markers of diffusion and are used by social media platforms, including Facebook’s algorithm to determine which social media content is shown to users and those in their network [35]. As a result, increasing user engagement has become a primary objective of social media interventions.

However, while many young adults interact with their friends and families on social media, many sexual health interventions have highlighted concerns about attrition and low engagement with sexual health information [36, 37], while others have attempted to increase user engagement through the use of paid advertisements [37, 38]. Since paid advertisements are not cost-effective and not sustainable, there is an increasing need to understand the predictors of user organic (unpaid) engagement, especially for potentially sensitive topics like sexual health information.

Despite this reality, it remains unclear whether and how young African adults use digital innovations like social media to access sexual health information. As a result, understanding the most successful tactics for reaching, engaging, and keeping young people in social media-based sexual health promotion, as well as the type of material that encourages user interaction or engagement, has become a higher priority. An awareness of these will definitely provide valuable insights for supporting successful online sexual health promotion campaigns and guide the design and development of context-specific social media content and the use of features that have a high appeal for young African adults [38].

### Predictors of engagement with health information on social media

An important consideration for effective sexual health education on social media is increasing user engagement, especially because more interactive social media content can increase exposure and ultimately better support behaviour change [34, 39]. Recent works looking at different social media users have found that most users tend to be passive, using social media to seek information or engage casually, keeping up with the online activities of others without sharing content or leaving an opinion [40]. As a result, a vast number of studies have examined multiple pathways to increase organic (unpaid) user engagement.

At the individual level, Olamijuwon & Odimegwu [31], using data from young African adults recruited via Facebook, highlight that young people were likely to access and interact with sexual health information on social media if such use improves their awareness of sexual and reproductive health and rights, is free of effort, and aligns with the way they interact with other information on social media. In their study, Andrade et al. (2018) also recommend that preventive messaging or other health promotion content should be strategically incorporated into habitual messages to keep participants connected with friends and ensure that the messages being conveyed resonate with their needs. Another study of top user profiles with high user engagement by Veale et al. [41] found that regular post updates directly engaging with users through individual responses and acknowledgements and encouraging interaction and conversation by posing questions were keys to successful engagement. Direct invitation for user engagement, such as asking users to comment or share a post, have also been associated with increased user engagement for comments and shares but not liking a post in another study [42]. On the other hand, asking a question was negatively associated with engagement [43].

Furthermore, the use of multimedia content is significantly associated with higher levels of user engagement [41]. This finding resonates with a prior study [37] and some recent studies [39, 42–44], all of which confirmed that multimedia content was positively increased engagement. Rus and Cameron [44], in their study of 10 diabetes-related Facebook pages, found that the use of imagery was the strongest predictor of user engagement, including liking and sharing a post. Goedel et al. [42] also found that posts containing photos, links, and videos received higher cumulative engagement scores than posts containing status updates only. After disaggregating by specific user engagement metrics, evidence from their study suggested that posts with links and photos received fewer comments status updates while posts with videos received higher engagement on all metrics [42]. Although combined with an advertisement, Pedrana et al. [45] also found that the webisode format of video uploads and the combination of education with entertainment was an effective driver of success in delivering health information to gay men. Adolescents in Tanzania also reported that peer-to-peer sharing of sexual health information would be higher if they were humourous [20]. Their findings altogether were consistent with the richness theory [46], suggesting that richer media content engages audiences better.

Beyond activity-based efforts to increase user engagement and content types, the content of messages and the expressions conveyed have also been shown to be associated with user engagement. Posts with positive affect elicit higher engagement, perhaps reflecting the well-documented heuristic bias towards positive messaging [44, 47]. In a similar study of 20 Facebook health profiles, Kite et al. [39] found that positive sentiments were significantly associated with high user engagement. Like the form of posts, the content of posts has also been documented to be associated with user engagement. A study of gay, bisexual and other men who have sex with men found that posts about pre-exposure prophylaxis and stigma exhibited greater engagement, while posts about dating exhibited lower levels of engagement [43]. This finding was also substantiated by a more recent study of HIV-related messages on Twitter, highlighting that fear-related language was a strong predictor of retweet counts on Twitter [48].

While many of the studies above offer an interesting yet diverse perspective on increasing user engagement, they are mostly limited in their approach and analysis. First is that most of the studies cited have used informatics-based methodology [42, 43, 48], which may not be very effective, as these approaches are subject to measurement error or obscure some data richness [49]. In addition, the use of automated analysis may only provide a partial understanding of the meanings embedded in a corpus of data and does not provide a profound insight into the context and processes that generated such discourse [50]. Other studies, on the contrary, have utilized paid advertisements to increase user engagement [37, 38] or focused on specific audiences such as the lesbian, gay, bisexual, and transgender [42] or gay and bisexual men [43]. In addition, many current studies have examined user engagement by creating an aggregate index that combines likes, shares and comments [41–43]. This approach implies that interaction and engagement metrics are weighted equally.

However, the relative merits of each engagement could vary according to educational objectives. For example, exposure to some messages could reap huge benefits, while others may need to promote interaction between educators and users [51]. As a result, it is increasingly becoming important to evaluate different metrics separately to enable better targeting of different strategies based on education objectives (to increase reach, or interaction or both). In addition, by summing up all metrics, researchers may be obscuring important relationships; notably, since interaction metrics may indicate different levels of engagement. For example, liking a post requires little cognitive effort than leaving a comment or sharing a post with other people in a user’s network. As a result, the predictors of superficial forms of engagement, such as liking a post, might be different from those requiring more cognitive efforts, such as leaving a comment [43], as observed by Goedel et al. [42].

In addition, most of the existing scholarship on the message properties that predicts organic user engagement are concentrated in developed countries, while scanty evidence abounds in developing countries, including Africa. The increasing adoption of social media platforms for sexual health education in African countries necessitates an urgent need to explore the predictors of engagement both from a global perspective and from the perspective of young adults since interventions addressing sensitive subjects and those targeting young people might be uniquely constrained by contexts and user willingness.

Furthermore, the extant scholarship has focused mostly on user engagement from the professional perspective, while very few studies have assessed user engagement from young people’s perspectives [20]. However, peer education may be more successful than adult-centred or didactic education in dealing with the sexual health challenges faced by young people. Given the huge gap in scholarship, there’s an urgent need for further analysis to document the type of participation that will best promote health awareness. This study builds on past research and leverages the latest technology on social media to understand the unique determinants of various types of engagement with peer-generated sexual health information.

### Current research

The present study aimed to (1) characterize peer-generated sexual and reproductive health information on a peer-led Facebook group that facilitates discussions about sexuality and reproductive health; (2) identify post features and content associated with greater user engagement. In achieving these objects, this study extends scholarship primarily in two ways. First, analyzing the different engagement metrics enable us to delineate the predictors of different types of user engagement. In addition, leveraging a platform where young people consume, produce and simultaneously interact with sexual health information provides a unique opportunity to understand the features of sexual health information that resonates and probably be compatible with young adults’ social media engagement habits.

Secondly, the use of human annotators enables us to identify additional features that predict user engagement while also considering the content and context of the messages. Furthermore, our analysis comes especially when there is a pressing need to digitize sexual health information and augment existing sexual health education strategies with digital innovations to minimize in-person contact. Therefore, the findings of this study will be of immense importance for the development of useful and engaging sexual health information to increase information dissemination and, ultimately, behavioural change.

## Methods

### Data source

Data for the study was obtained from a youth-led Facebook group that facilitates discussion about sexuality among young African adults. We focused on data from Facebook because Facebook is the most popular and frequently used social media platform by many people of various ages worldwide, including Africa [52, 53]. The group was created in 2016 by an individual and had over 25 content moderators, mostly young adults from different African countries. The group is also public, implying that anyone could find the group or join the group, see who is in the group and what is posted on the group. Facebook users who find the group can self-select to join the group or be invited by anyone already in the group.

The group has 176,461 members, mostly from African countries and contains several messages on intimate relationships, dating and marriage. A summary description of the group demographics is presented in Table 1. About 91% of the group members live in Nigeria, with a lower percentage of participants from other African countries, including Ghana (0.6%), South Africa (0.4%), Kenya (0.3%), and the Republic of Benin (0.3%). About 51% of the group members are women, while adolescents and young adults account for about 71% of the group membership. The demographic diversity of participants in the group and the diversity of the messages posted on the group make it a valuable resource for studying how young African adults interact with peer-generated sexuality information.

**Table 1:** Group demographics as at May 31, 2019 *(Source: Facebook Group Insights)*

Characteristics	Number of Members	Percentages (%)
**Country**		
Nigeria	160,037	91.00%
Ghana	989	0.56%
South Africa	692	0.39%
Benin	442	0.25%
Kenya	434	0.25%
Other African	2229	1.27%
Non-African	10,835	6.16%
Country unknown	200	0.11%
**Gender**		
Women	90,096	51.23%
Men	85,737	48.75%
Unknown	25	0.01%
**Age**		
13–17	1812	1.03%
18–24	64,978	36.95%
25–34	68,853	39.15%
35+	40,215	22.87%
	175,858	100.0%

### Text sample

A data scrapping application (Sociograph.io) was used to download public wall posts on the Facebook group. The application was installed directly on the Facebook group with administrative rights so that it could access all messages and interactions. Permission to access public wall posts on the group was obtained from the group creator after receiving a detailed description of the study and its potential for sexual and reproductive health intervention.

As with other studies using social media data from existing groups, obtaining informed consent from all the group members was practically impossible [50]. However, we made conscious efforts to mitigate risks against research subjects and the group. First, all the data extracted contained no personal identifying characteristics, such as the name of the content creator. However, since some content creators on the group included their information (such as mobile numbers, links to other groups, names and email addresses) on public wall posts shared on the group, we carefully examined each post and removed these during data coding. This strategy aligns with recommendations in the literature [54, 55]. We also rephrased quotes included in our study to minimize the identification of posts through a keyword search on the social media platform.

A total of 62,986 member-generated posts and 897,967 comments made since 2017 were retrieved from the group. These wall posts include messages related or not related to sexuality since the members of the group could post any messages on the group regardless of whether they focus specifically on sexuality. Our data analysis and management followed a two-stage process. In the first stage, we developed a keyword-based dictionary comprising words related to several aspects of sexual and reproductive health such as “condom,” “contracept,” “rape,” “virgin,” “sex,” and “s*x,” among several others. A full list of the keywords is presented in Table 2. We included these words in the dictionary based on their popularity and potential to fully capture all relevant sexuality messages. The keyword-based filtering looped through each corpus of messages and retained only posts that included at least one of the keywords. We also did not find any relevant message that had already not been captured by any of the keywords during data validation. With the keyword-based filtering, we identified a total of 8497 posts that had at least one or more keywords.

**Table 2:** Full list of keywords

Keywords				
erection	condom	hiv	violence	boyfriend
masturbat	contracep	aids	beating	girlfriend
sex	abortion	std	rape	dating
s*x	pregnant	stis		lesbian
virgin				gay

The 8497 posts identified were reviewed by seven research assistants who reviewed an average of 2550 Facebook posts each over two weeks. This implied that two research assistants reviewed each post. The first author reviewed the remainder of the posts. Each assistant was asked to classify the posts based on whether they related to a set of predefined sub-domains of sexual and reproductive health, including sexual abstinence and contraceptive use, among others. The assistants were trained extensively before the activity. About 54% of the posts were deleted if two assistants classified the posts as not being related to sexual and reproductive health. The final dataset during this phase comprised 3947 posts shared on the group between June 1, 2018, and May 31, 2019.

### Metadata

Metadata represents supplementary information—data about the data—that is embedded in each Facebook post on the group and included in the corpus of data collected. These metadata include a postID (a unique numerical identifier assigned to each Facebook post), the post date, post type (status/link/photo/video) and measures of interactions and propagation such as reactions (like/angry/haha/love), comments and shares. In addition to coded categories, these data were used to describe the interactions with sexual and reproductive health information on the group.

### Coding

To develop an initial coding system, we used past definitions of sexuality education to identify four unique sexual and reproductive health topics: abuse, birth control and abortion, intimate relations, and sexual abstinence [56–59]. After an extensive review of the literature [58, 60], we further classified a group of messages about abstaining from all non-penetrative sexual activities (or (not) having sexual feelings such as self-pleasure touching, and kissing) as sexual purity while messages related to penetrative sexual activity (primarily before marriage) as sexual abstinence. This description is also consistent with what is known in literature and what young adults define as sex [61]. The final codebook included coding categories, all with definitions, and examples are presented in Table 3.

**Table 3:** Coding scheme and frequencies of sexuality education topics, strategy, and tone of communication

Classification	Description of Posts	Excerpted Exemplars of Posts	Freq	Percentage
**TOPIC**				
Abuse	Posts related to all forms of violence, including emotional violence, physical or sexual violence.	The rates at which rape is becoming prevalent and ending the dream of some girls from being virgins till marriage is very discouraging and disturbing. Some of the wicked rapists either rape for ritual purposes or because of their lack of sexual discipline.	130	3.7%
Birth Control and Abortion	Posts about the use or non-use of birth control methods and abortion to prevent/delay a marital or premarital birth. This category also includes posts about all matters related to getting pregnant.	Dear sisters, just because he proposed marriage does not mean that you should begin the use of contraceptives.	38	1.1%
Intimate Relations	Posts about relationships/dating/union dynamics. These also include posts about how to attract a “good” partner or the process of preparing for marriage/courtship/relationships.	Someone asked if the ladies in this group can marry a man living in a room apartment. I was shocked to see responses like “I can’t.” No one wishes to be poor, but will you refuse a man’s marital advances he is not wealthy?	1959	55.4%
Sexual Abstinence	Posts about sex or sexual intercourse before marriage as well as its consequences (premarital pregnancy or birth).	Dear Lord, may all singles who are planning to have sex on Valentine’s Day get pregnant, let no abortion pill work for them, may all condoms tear miraculously, do not let any emergency pill work for them.	1018	28.8%
Sexual Purity	Posts about being decent in dressing, avoiding sexual urge/feelings, nor alternative means of sexual intimacy including masturbation, smooching, kissing, hugging or other erotic activities but not sex.	Kissing and other foreplay prepare the body for sex. If you really do not want sex, as you claimed, stay clear until marriage!	388	11.0%
**STRATEGY**				
Status Update	General status messages that are intended to motivate behaviour change without telling a story, sharing an experience, or seeking an opinion.	Dear ladies, if you’re in a relationship with a man who doesn’t respect you, fights with you, or makes you feel less than yourself because you don’t want to have sex with him before marriage, he’s not your Mr. Right! Don’t stay in that toxic relationship! It’s not going to be worth it! Keep your head held high as you leave.	2594	73.4%
Experience sharing	Sexual health messages on the group based on an author’s personal experience.	There was a time I was struggling with masturbation, and I got very frustrated because I was really struggling spiritually. Guilt and condemnation haunted me until one day when I asked myself why I was masturbating? That day I got my freedom from masturbation.	81	2.3%
Request for opinion	Posts in which the author is seeking advice about a sexual and reproductive health/rights issue or intimate relations.	Can someone be deflowered by having sex?	351	9.9%
Storytelling	Posts that tell a story - often in episodes.	“Ben raped me. How could he? I thought he was a Christian, but now I know better.” Beatrice sobbed uncontrollably. She recalled the actions that led to the wanton molestation.	507	14.4%
**TONE**				
Fear	Persuasive wall posts that emphasize the potential danger and harm that will befall members of the group if they engage in a specific behaviour do not adopt the messages’ sexual or reproductive recommendations.	Dear boys and girls, condoms may protect you from sexually transmitted diseases but not from Spiritual Transmitted Demons (STDs).	331	9.4%
Guilt	Wall posts that evoke feelings of guilt - feelings like why have/did I done/did this. This also includes posts that blame an individual for the negative experience of another.	Young lady, do you believe you are helping yourself by seeking to kill that precious baby through abortion? Would you have been able to commit the sin that led to the undesired pregnancy you are carrying if your mother had terminated you? It’s not a good idea. It is deadly.	373	10.6%
Neutral	Posts that are conveyed in a manner that is void of guilt, stigma or fear.	If you have sex in the dream, it’s not necessarily implying that you have a spiritual husband or wife. It’s warning you to watch how much attention you give to sex and romantic thoughts.	2718	76.9%
Stigma	Posts that are shared on the group with the intention of shaming or stigmatizing people who engage in a particular behaviour.	If you are not a virgin on your wedding day, you are not supposed to hold a bouquet because you are no longer a virgin. Holding a flower is a symbol of virginity.	111	3.1%
**TYPE**				
Text only	Posts that does not include a link or photos or videos		2730	77.3%
Link	Posts that includes only a link but without a photo or video		26	0.7%
Photo or video	Posts that includes a video or a photo		777	22.0%

Note: Quotes have been rephrased to anonymize the group, and all the spelling, grammar, and punctuation errors of the original post have been addressed

We further engaged six of the initial research assistants to review the 3947 Facebook messages classified to be related to several aspects of sexual and reproductive health. All six assistants were young adults aged 20–25 years and were university students. The assistants were re-trained and asked to classify the Facebook posts based on topic, strategy, and tone of communication. An average of 1315 messages was reviewed and classified by each of the six assistants during this phase. Krippendorff’s alpha for topic classification (α = 0.86), strategy (α = 0.91) and tone (α = 0.73) of communication was above the acceptable minimum levels of reliability [62]. The first author also reviewed a random sample of 500 messages in the dataset to ensure consistency and accuracy in data coding.

### Analysis

Content analysis was used to examine the messages shared on the Facebook group to assess how young adults interacted with the different sub-domains/topics of sexual and reproductive health, type and tone of message communications. Quantitative content analysis involves the systemic analysis of texts or symbols of communication that have assigned numeric values based on a predefined coding scheme [63]. It also involves analyzing relationships among the assigned numeric values using statistical methods to describe the communication and draw valid inferences from the texts to its context [63].

In our investigation, we adopted several measures to answer our main research question. We also examined interactions and propagation of messages on the group using the three counts of engagement comprising of “reactions” (like, love, sad, and angry), “comments,” and “shares,” all of which are considered as aspects of “electronic word-of-mouth” and could stimulate different communication behaviours [64, 65]. We also assessed the bivariate relationships between the different topics, messaging strategy and the tone of communication.

Finally, we fitted four negative binomial regression models using the **glm.nb** function from the MASS package [66] to delineate statistically significant differences in young adults interacting with each of the messages by topic, type, strategy, and tone of communication. This model was chosen because the data were highly skewed and overdispersed [67]. We ran four models, one for each measure of message interaction and propagation: Model 1 considered associations between the sum of all interactions and each of the message metadata. Model 2 considered the associations between the number of reactions and each of the message categories. Model 3 considered the association between the number of comments and each of the message categories. Finally, Model 4 considered the association between the number of comments and each of the message categories. Interpretation of the results was made using incidence rate ratios (IRR) and 95% confidence intervals. An incidence rate ratio greater than one (IRR > 1) implied a higher likelihood, while incidence rate ratios less than one (IRR < 1) implied a lower likelihood, and incidence rate ratios equal to one (IRR = 1) implied no difference in risks. All data analyses were conducted in R [68]. De-identified data supporting this study’s findings and reproducible R codes for tables, all graphs and negative binomial model outputs are available online. Ethical approval for the study was obtained from the non-medical human research ethics committee at the University of the Witwatersrand.

## Results

### Characterizing peer-communicated sexuality information by topic, strategy, type and tone of communication

Table 3 summarises messages shared on the group by topic classification, strategy, and tone. Most of the messages on the group were about relationships and dating, while about 40% of the messages were on sexual abstinence or purity. Regarding messaging strategy, close to one-quarter of the messages were regular status updates, while about 10% of the messages requested opinions, and about 14% told a story. The tone of about one-quarter of the messages evoked fear, stigma, or guilt. Lastly, most messages on the group are text-only posts (does not include any multimedia or link), while about 22% of messages on the group included at least a photo or video.

**Fig. 1:**
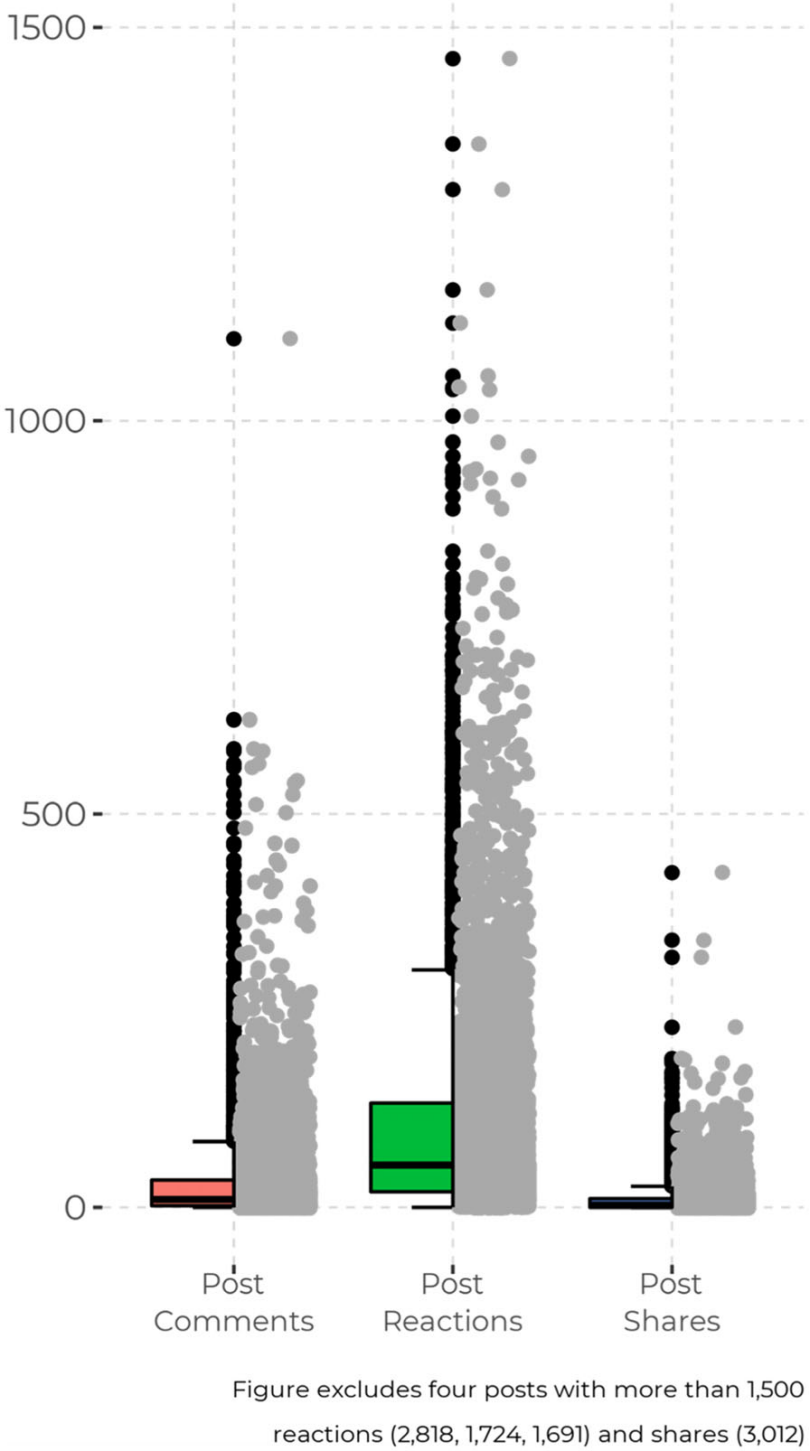
Levels of interaction and propagation of peer-communicated sexuality information in the sample

We examined significant differences in the tone and strategy for which different topic classifications were communicated in Fig. 1. The results showed that about 46% of messages on abuse were telling a story compared to less than one-quarter among birth control and abortion-related messages (21%), intimate relations (14%), sexual abstinence (11%), and sexual purity (12). Requests for opinions were more common among family planning messages (13%) and sexual abstinence (12%). Furthermore, topic classification across tone showed that more than half of family planning messages evoked fear (26%), stigma (5%), or guilt (21%). Similarly, 41 and 33% of sexual abstinence and purity messages evoked fear, stigma, or guilt.

### Interaction and propagation of peer-communicated sexuality information

**Fig. 2:**
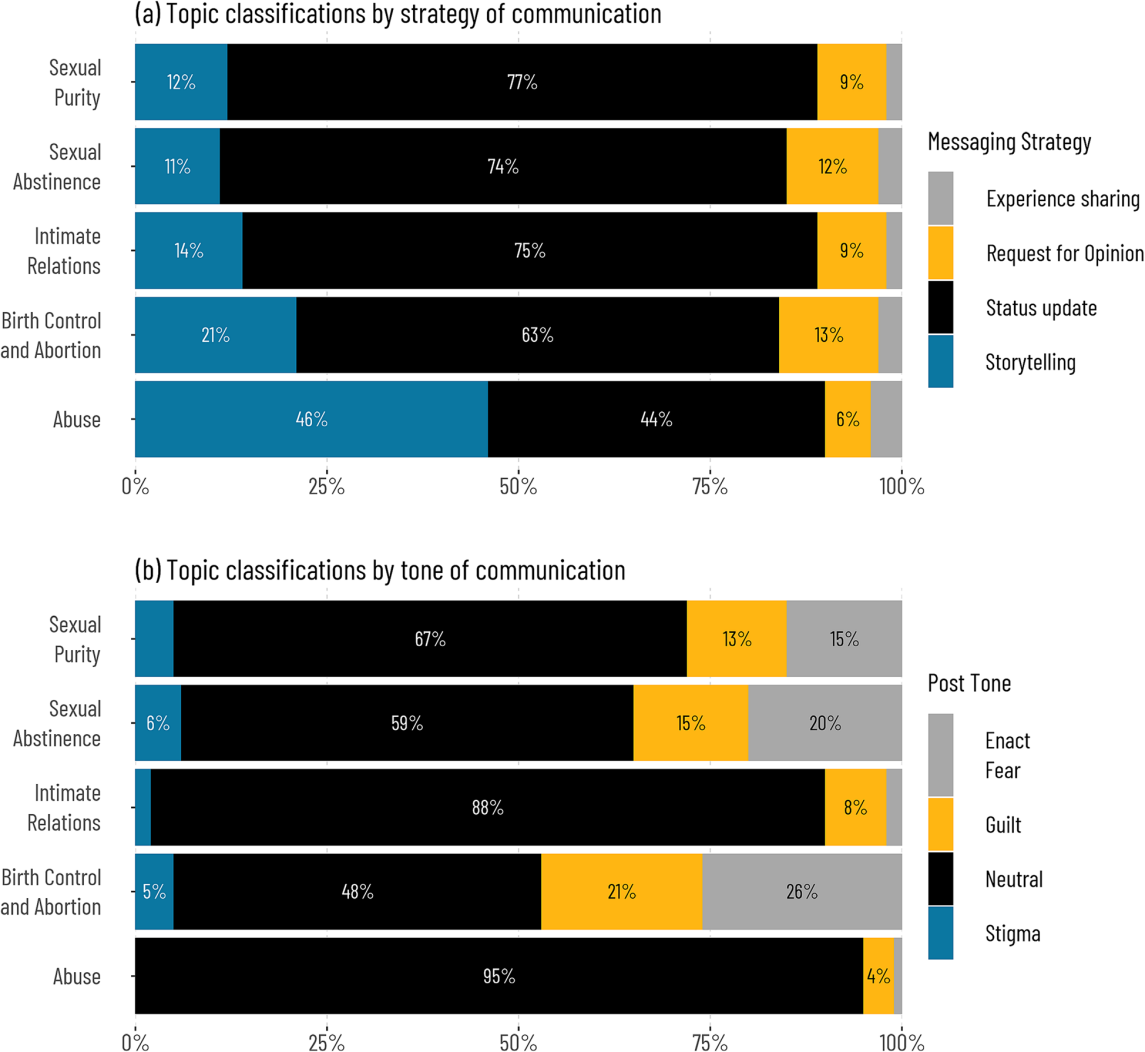
Classification of the peer-communicated sexuality information by topic, strategy, and tone of communication

The level of interaction for all sexual health information shared on the group is presented in Fig. 2. The median level of interaction was highest for reactions (x̃ = 54) and comments (x̃ = 10) but lower for post shares (x̃ = 10). Table 4 presents the results of the multiple negative binomial regression model testing associations between the measures of message interaction and propagation. The results showed that interactions with messages on abuse were significantly different from interactions with messages on intimate relations in terms of the number of reactions and shares. Members of the group were significantly more likely to react to [IRR: 1.14, 95% CI: 1.04–1.24], and comment on [IRR: 1.26, 95% CI: 1.12–1.43], sexual abstinence-based messages compared to messages on intimate relations. Compared to messages on intimate relations, those in the group were significantly less likely to share messages on abuse [IRR:0.34, 95% CI: 0.26–0.46], sexual abstinence [IRR: 0.56, 95% CI: 0.49–0.63], and sexual purity [IRR: 0.57, 95% CI: 0.48–0.69].

**Table 4:** Associations between message features and the indices of message interaction and propagation

Characteristics	Total Interactions		Number of Reactions		Number of Comments		Number of Shares	
	Incidence Rate Ratios (IRR)	95% CI	Incidence Rate Ratios (IRR)	95% CI	Incidence Rate Ratios (IRR)	95% CI	Incidence Rate Ratios (IRR)	95% CI
**Topic**								
Intimate Relations	Reference		Reference		Reference		Reference	
Abuse	0.73**	0.6–0.91	0.75**	0.62–0.92	0.9	0.69–1.2	0.34***	0.26–0.46
Birth Control and Abortion	0.87	0.62–1.29	0.88	0.63–1.28	0.89	0.55–1.55	0.83	0.50–1.49
Sexual Abstinence	1.11*	1.02–1.22	1.14**	1.04–1.24	1.26***	1.12–1.43	0.56***	0.49–0.63
Sexual Purity	0.98	0.86–1.11	1.00	0.89–1.13	1.02	0.86–1.22	0.57***	0.48–0.69
**Tone**								
Neutral	Reference		Reference		Reference		Reference	
Fear	0.78***	0.68–0.9	0.75***	0.66–0.86	0.81*	0.67–0.99	1.21	1.00–1.47
Guilt	0.84**	0.74–0.95	0.82**	0.72–0.92	0.94	0.80–1.12	0.99	0.83–1.18
Stigma	1.17	0.95–1.47	1.12	0.92–1.4	1.51**	1.13–2.06	1.21	0.91–1.66
**Strategy**								
Status Update	Reference		Reference		Reference		Reference	
Experience sharing	2.22***	1.74–2.88	2.25***	1.78–2.89	2.46***	1.77–3.53	1.72**	1.24–2.47
Request for opinion	1.10	0.97–1.26	0.48***	0.43–0.55	4.25***	3.57–5.1	0.13***	0.11–0.16
Storytelling	1.46***	1.31–1.63	1.42***	1.28–1.59	1.69***	1.45–1.96	1.5***	1.29–1.75
**Type**								
Text only	Reference		Reference		Reference		Reference	
Link	0.18***	0.12–0.29	0.17***	0.11–0.27	0.16***	0.09–0.31	0.34***	0.19–0.67
Photo	1.64***	1.5–1.8	1.76***	1.61–1.92	1.16*	1.02–1.32	1.91***	1.68–2.16
Observations	3533		3533		3533		3533	
Log Likelihood	−21,049.05		−19,789.82		−14,468.14		−10,931.93	
Akaike Inf. Crit.	42,124.09		39,605.65		28,962.28		21,889.87	

Note: *** *p* < 0.001, ** *p* < 0.01, * *p* < 0.05; *CI* Confidence interval; *IRR* Incidence Rate Ratio

Messages that evoked fear [IRR:0.75, 95% CI: 0.66–0.86] or guilt [IRR:0.82, 95% CI: 0.72–0.92] received significantly fewer reactions compared to neutral messages. Messages that evoked fear [IRR: 0.81, 95% CI: 0.67–0.99] also received a significantly fewer number of comments, while stigma based messages [IRR: 1.55, 95% CI: 1.13–2.06] received a significantly higher number of comments compared to neutral messages. No statistically significant differences were observed in the propagation (number of shares) of messages by the tone of communication.

Sharing an experience or telling a story were associated with a higher number of reactions and comments compared to counselling. Messages requesting an opinion also had a significantly higher number of comments [IRR: 4.25, 95% CI: 3.57–5.10] but a lower number of reactions [IRR: 0.48, 95% CI: 0.43–0.55] and shares [IRR: 0.13, 95% CI: 0.11–0.16] compared to counselling messages. Including a photo in messages was associated with a higher number of reactions [IRR: 1.76, 95% CI: 1.61–1.92], comments [IRR: 1.16, 95% CI: 1.02–1.32] and shares [IRR: 1.91, 95% CI: 1.68–2.16] compared to text-only messages. Including a link, on the other hand, was associated with a lower number of reactions [IRR: 0.17, 95% CI: 0.11–0.27], comments [IRR: 0.16, 95% CI: 0.09–0.31], and shares [IRR: 0.34, 95% CI: 0.19–0.67] compared to text-only messages.

## Discussion

This study is one of the first to document the actual use of Facebook as a resource for peer communication of sexual health information and intimate relations among young African adults. Some notable findings from our data highlighted three critical areas of consideration in designing effective and engaging sexual and reproductive health information for young African adults. First, assessing the levels of engagement based on the different metrics suggested that young people were more likely to engage superficially with peer-generated sexual health messages using reactions than leaving comments or sharing messages with those in their network. In addition, our analysis showed that compared to messages on intimate relations, young adults were significantly less likely to share sexual abstinence and purity-based messages with their network. Although our study did not explore in detail the content of the messages shared in this network, concerns about anonymity and privacy may have affected the propagation of these messages. This finding resonates with prior studies suggesting that young adults may be less likely to interact with sensitive topics such as sexual and reproductive health information to circumvent monitoring by parents and friends [69–72].

Secondly, our analysis also revealed the use of visual content and multimedia in communicating sexual health information. Consistent with a recent study on HIV information dissemination on Twitter, we found that young people were more likely to interact with messages with visual or multimedia content but less likely to interact with messages with links [48]. This finding also resonates with prior studies [37, 39, 43, 44] and the richness theory [46], all of which affirmed the positive association between the use of rich message features like multimedia and increased user engagement. Young adults likely find it easier to comprehend messages with multimedia content and may ignore messages including a link due to the financial effort and time required to visit the link and understand the messages [48]. Interestingly, our data revealed that young adults were more likely to interact with messages telling a story or sharing an experience. When asked for their opinions, young adults were also more likely to comment. This approach may be useful in getting young adults’ perspectives about issues of importance.

Furthermore, the preponderance of messages on intimate relationships, sexual abstinence particularly until marriage, and purity-based messages on the group highlight how sexuality messaging have been reduced to diseases, danger and a response to the HIV/AIDS epidemic— a pattern that is consistent with the literature [73, 74]. Precisely, there is a dominance of a precautionary voice and a language of consequence, in which young people on the group are advised to abstain from sexual practices or ‘face consequences’ [75, 76] of HIV infection, sexually transmitted infections (STIs) and pregnancy. This finding is further corroborated by the significant amount of fear-based, stigma, and guilt appeal messages. Precisely, we observed that close to one-quarter of the sexuality information on the group evoked fear, stigma or guilt, with variations as high as more than half among family planning messages and more than one-quarter of sexual abstinence and purity messages.

This pattern of fear, shame and blame tactic is particularly of great concern given that young people who should be key for delivering comprehensive and less threatening sexuality information are observed to be reinforcing existing dominant binary gender roles, norms and moralistic positions on young people’s sexuality. Evidence of the use of fear appeals and “scare tactics” in sexual and reproductive health messages have been observed in the African literature [77–80]. In one study of parent-led sexual health information, it emerged that parents deliberately misinform their children about sexuality using fear-based appeals, stigma and guilt focusing themes that depict young people’s sexual behaviours in terms of deviance, immorality, and waywardness as approaches to regulating young people’s awareness and knowledge of sexual and reproductive health [77].

Sexuality education in South Africa and indeed much of Africa also appears to be dominated by negative connotations of sexuality through the narratives of sickness, danger, doomed futures, violence, and repercussions [79]. For example, horrific depictions of sexually transmitted diseases and premarital childbearing have reportedly been used to frighten students into abstinence [80]. These negative connations of sexuality are primarily because of the dominant misconception that sexual health education will inspire sexual imaginations in young people [77, 81, 82]. These approaches of sexual health communication are further rooted in religious teachings, and cultural attitudes that promote sexual abstinence-only until marriage but which altogether does not address the sexual health challenges of young people nor are effective in motivating behavioural change [83–87]. Precisely, Bhana et al. [88] emphasized that sexual health interventions that emphasize ‘risk’ over ‘desire’ and ‘shame’ over ‘pleasure’ risk speak to no one, especially young people whose bodies and experiences tell them otherwise.

Till today, the role of fear, guilt, and stigma appeals in behavioural change has yielded mixed findings and is contested both by researchers and practitioners [81, 82, 89–91]. Some studies have shown that the use of fear appeals in messages generates strong responses and may indeed increase awareness of the hazards of unhealthy practices, influencing attitudes, intentions, and ultimately behavioural changes [90]. For example, fear appeals in HIV prevention messages during the early period of the national response to the HIV epidemic are believed to have contributed to an approximate 66% decline in HIV prevalence in Uganda [82, 91]. The use of fear appeals in sexual health education has also been observed to have no adverse consequences or undesirable outcomes on the population they intend to target [90]. However, other studies contest that even when fear appeals arouse the interest of those who are exposed to them, they are often not associated with health behaviour changes [92]. Others argue that by inundating young adults with fear, they fail to seek out information on reducing their risk or finding out whether they are infected [77, 81].

Surprisingly, young adults on our peer-led Facebook group were less likely to interact with messages with fear and guilt appeals. However, this finding contrasts with prior literature on the dissemination of sexual health information on social media [48, 93, 94]. Most of these studies were focused on HIV prevention messages on Twitter, and they had a different audience compared to the present study. Nonetheless, the low level of interaction with non-neutral messages is likely a sign of disapproval of these messages and the hesitance to propagate the same while also leaving comments to highlight these concerns.

Finally, our study contributes to the literature in important ways, but it is not without limitations. The first is that we attempted to utilize data that was cross-cutting throughout Africa. However, about 90% of the members of the Facebook group are from Nigeria. As a result, the discussions on the group may have been dominated by voices from Nigeria, where religion and culture are known to play pivotal roles in sexual health communication. Nonetheless, the role of religion and culture in sexual health promotion has also been observed in other African countries like South Africa, Uganda, and several others.

Furthermore, we retrieved wall posts and comments from only one Facebook group. As a result, the generalizability of our findings is limited. Nevertheless, we found similar associations as did previous studies and identified new predictors of user engagement. While we focus on data from Facebook, we believe that many of the findings, especially those involving the use of rich messages features, will extend to other platforms, including multimedia social media sites like Instagram. While our data set comprises a significant number of meaningful discourses about sexuality education for and by young African adults, these messages on the group did not represent all such discourse. Most significantly, we observed an abundance of sexual abstinence and purity-based messages. As a result, the findings should be interpreted with caution. It is also worthy of note that the level of interactions and propagation of messages on the group may be influenced by author popularity. For example, young adults may interact with messages from authors who are well known in the group. However, our data do not include author profile details, so we could not examine this association. Despite the limitations, our study provides insight that could be useful when considering social media for sexual health communication among young African adults.

## Conclusion

We examined the use of Facebook for peer-communication of sexuality information as well as how young African adults interact with these. We found that young adults were more likely to superficially interact with peer-communicated sexual health information through likes than engage (comments) or propagate such messages. The use of storytelling and the inclusion of multimedia such as photos or videos was associated with higher levels of interaction with peer-communicated sexuality information. On the other hand, the use of fear appeals in sexuality information was associated with lower levels of interaction through comments and likes. These findings provide valuable insight and pave the way for the design of useful, engaging, and context-specific sexual health information that uses features that have a high appeal for young African adults and advance the sexual and reproductive health of young African adults.

The data derived from our analysis can be used to complement policy design in developing tailored participatory sexual health promotion for young African adults. This opportunity may help empower the sexual and reproductive health rights of young African adults and reduce some of the misinformation that impedes progress. Since our analysis did not explore what young adults are doing with the information, particularly those that evoked fear, guilt, or stigma, future studies could examine this and how young adults respond to these messages. Most importantly, future studies are needed to explicate whether the level of engagement with fear-based sexual health information on social media are correlated with the perceived efficacy and if such messages motivate significant behavioural changes as demonstrated in the theories of fear-based [90, 95].

## Data Availability

De-identified data that supports the findings of this study and reproducible R codes for tables, all graphs and negative binomial model outputs are available online: https://osf.io/bzyu6/

## Abbreviations

HIV: Human Immunodeficiency Virus
IRR: Incidence Rate Ratios
95% CI: 95% Confidence Intervals
